# 1-Meth­oxy-3-*o*-tolyl­bicyclo­[2.2.2]oct-5-ene-2,2-dicarbonitrile

**DOI:** 10.1107/S1600536809032474

**Published:** 2009-08-22

**Authors:** Orhan Büyükgüngör, Serkan Yavuz, Mustafa Odabaşoğlu, Özgür Pamir, Yılmaz Yıldırır

**Affiliations:** a Department of Physics, Faculty of Arts & Science, Ondokuz Mayıs University, TR-55139 Kurupelit Samsun, Turkey; b Department of Chemistry, Faculty of Arts & Science, Gazi University, Ankara, Turkey; c Chemical Technology Program, Denizli Higher Vocational School, Pamukkale University, TR-20159 Kınıklı, Denizli, Turkey

## Abstract

In the title compound, C_18_H_18_N_2_O, the cyclo­hexene and cyclo­hexane rings of the bicyclo­[2.2.2]oct-5-ene unit adopt distorted boat conformations. In the crystal, mol­ecules exist as C—H⋯N hydrogen-bonded centrosymmetric *R*_2_^2^(14) dimers, which are further linked by C—H⋯π inter­actions.

## Related literature

For general background, see: Çete *et al.* (2007 ▶); Corey (2002 ▶); Kurt & Anker (1998 ▶); Mamedov *et al.* (2007 ▶); Özkan *et al.*, (2007 ▶); Potapov (1988 ▶). For the synthesis, see: Zhang *et al.* (2006 ▶). For graph-set notation, see: Bernstein *et al.* (1995 ▶); Etter (1990 ▶). For ring conformations, see: Cremer & Pople (1975 ▶).
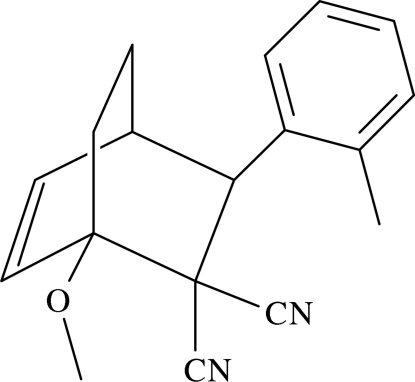


## Experimental

### 

#### Crystal data

C_18_H_18_N_2_O
*M* *_r_* = 278.34
Triclinic, 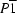

*a* = 7.5922 (6) Å
*b* = 9.5026 (8) Å
*c* = 11.5584 (9) Å
α = 91.201 (7)°
β = 107.206 (6)°
γ = 110.856 (6)°
*V* = 736.89 (10) Å^3^
*Z* = 2
Mo *K*α radiation
μ = 0.08 mm^−1^
*T* = 296 K
0.48 × 0.42 × 0.17 mm

#### Data collection

Stoe IPDS II diffractometer
Absorption correction: integration (*X-RED32*; Stoe & Cie, 2002 ▶) *T*_min_ = 0.956, *T*_max_ = 0.989
8047 measured reflections
3057 independent reflections
2532 reflections with *I* > 2σ(*I*)
*R*_int_ = 0.034

#### Refinement

*R*[*F* ^2^ > 2σ(*F* ^2^)] = 0.041
*wR*(*F* ^2^) = 0.108
*S* = 1.05
3057 reflections
192 parameters
H-atom parameters constrained
Δρ_max_ = 0.21 e Å^−3^
Δρ_min_ = −0.16 e Å^−3^

### 

Data collection: *X-AREA* (Stoe & Cie, 2002 ▶); cell refinement: *X-AREA*; data reduction: *X-RED32* (Stoe & Cie, 2002 ▶); program(s) used to solve structure: *SHELXS97* (Sheldrick, 2008 ▶); program(s) used to refine structure: *SHELXL97* (Sheldrick, 2008 ▶); molecular graphics: *ORTEP-3 for Windows* (Farrugia, 1997 ▶); software used to prepare material for publication: *WinGX* (Farrugia, 1999 ▶).

## Supplementary Material

Crystal structure: contains datablocks I, global. DOI: 10.1107/S1600536809032474/ci2877sup1.cif

Structure factors: contains datablocks I. DOI: 10.1107/S1600536809032474/ci2877Isup2.hkl

Additional supplementary materials:  crystallographic information; 3D view; checkCIF report

## Figures and Tables

**Table 1:** Hydrogen-bond geometry (Å, °)

*D*—H⋯*A*	*D*—H	H⋯*A*	*D*⋯*A*	*D*—H⋯*A*
C12—H12⋯N1^i^	0.93	2.70	3.509 (3)	146
C7—H7*C*⋯*Cg*1^ii^	0.96	2.84	3.688 (2)	146

Symmetry codes: (i) 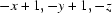; (ii) 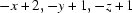. *Cg*1 is the centroid of the C1–C6 ring.

**Fig. 1.**
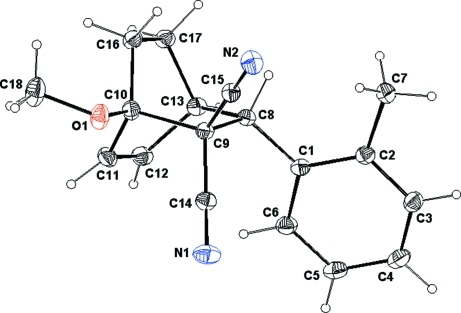
The molecular structure of (I), with the atomic numbering scheme. Displacement ellipsoids are drawn at the 15% probability level.

**Fig. 2.**
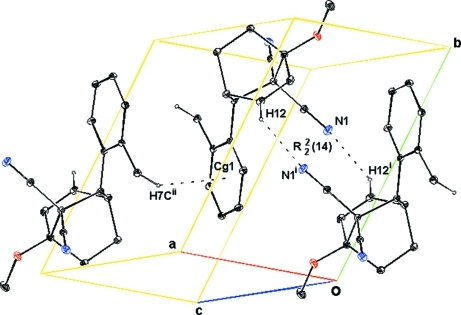
Part of the crystal structure of (I), showing the formation of R22(14) dimers and a C—H···π interaction. H atoms not involved in the interactions have been omitted for clarity. The dashed lines indicate hydrogen bonds. [Symmetry code: (i) 1 - x, 1 - y, -z; (ii) 2- x, 1 - y, 1 - z]. Cg1 is the centroid of the C1-C6 ring.

**Fig. 3.**
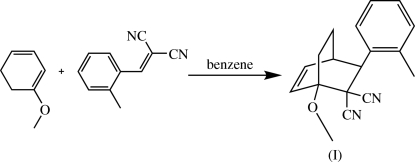
Preparation of the title compound.

## supplementary crystallographic information

### Comment

The Diels-Alder reactions are among the most useful of all synthetic processes for the construction of complex molecules and, for this reason, they have been studied extensively (Kurt & Anker, 1998). The reaction is easy, rapid and is a key reaction in fundamental organic synthesis. Cycloadducts of asymmetric Diels-Alder reactions have attracted attention owing to their utility in the synthesis of natural compounds (Corey, 2002).

In the conventional Diels-Alder reaction a double bond adds 1,4 to a conjugated diene. The title compound, (I), was prepared by a cycloaddiction reaction from 2-(2-methylbenzylidene) malononitrile and 1-methoxycyclohexa-1,3-diene. Bicyclo[2.2.2]octane and bicycle[2.2.2]octane moieties are essential fragment of many important natural and synthetic biologically active compounds (Potapov, 1988). Both this type bicyclo compounds and many cyano group containing compounds show biological activity (Özkan *et al.* 2007; Çete *et al.* 2007). Therefore, synthesis of these compounds in the practically active form is of practical interest (Mamedov *et al.* 2007).

The overall view and atom-labeling of the molecule of (I) are displayed in Fig. 1. The hydrogen-bonding parameters are given in Table 1 and the packing arrangement of the molecules is illustrated in Fig. 2. In the molecule, cyclohexene rings A(C8/C9/C10/C11/C12/C13) and B(C10/C11/C12/C13/C17/C16), and the cyclohexane ring C(C8/C9/C10/C16/C17/C13) of the bicyclo[2.2.2]oct-5-ene unit all adopt distorted boat conformations. The Cremer and Pople (1975) puckering parameters Q, θ and φ are 0.810 (2) Å, 84.8 (1)° and 111.8 (1)°, respectively for ring A, 0.788 (2) Å, 86.7 (1)° and 186.3 (1)°, respectively for ring B, and 0.906 (2) Å, 88.4 (1)° and 310.3 (1)°, respectively for ring C.

The crystal structure is stabilized by intermolecular C—H···N hydrogen bonds and C—H···π interactions (Table 1). As shown in Fig. 2, the molecules exist as C12—H12···N1 hydrogen-bonded centrosymmetric *R*_2_^2^(14) dimers (Bernstein *et al.*, 1995; Etter, 1990). The dimers are linked through C7—H7C···π interactions.

### Experimental

2-(2-Methylbenzylidene)malononitrile was prepared from 2-methyl benzaldehyde, malononitrile and potassium carbonate according to the literature method (Zhang *et al.* 2006). For the preparation of the title compound, 1-methoxycyclohexa-1,3-diene (330 mg, 3 mmol) and 2-(2-methylbenzylidene) malononitrile (459 mg, 3 mmol) were dissolved in benzene (20 ml). The reaction mixture was refluxed for 4 h, and monitored by TLC. After evaporation of the solvent, the reaction mixture was separated by column chromatography, using the mixture of hexane-ethyl acetate (1:2) as the eluant. The title compound was recrystallized from methanol in 3 d (m.p. 431–432 K).

### Refinement

H atoms were positioned geometrically (C-H = 0.93–0.98 Å) and refined using a riding model with *U*_iso_(H) = 1.2*U*_eq_(C) and 1.5*U*_eq_(methyl C). A rotating–group model was used for the methyl groups.

### Crystal data

**Table:**

C_18_H_18_N_2_O	*Z* = 2
*M**_r_* = 278.34	*F*(000) = 296
Triclinic, *P*1	*D*_x_ = 1.254 Mg m^−^^3^
Hall symbol: -P 1	Mo *K*α radiation, λ = 0.71073 Å
*a* = 7.5922 (6) Å	Cell parameters from 8047 reflections
*b* = 9.5026 (8) Å	θ = 1.9–28.1°
*c* = 11.5584 (9) Å	µ = 0.08 mm^−^^1^
α = 91.201 (7)°	*T* = 296 K
β = 107.206 (6)°	Prism, colourless
γ = 110.856 (6)°	0.48 × 0.42 × 0.17 mm
*V* = 736.89 (10) Å^3^	

### Data collection

**Table:**

Stoe IPDS II diffractometer	3057 independent reflections
Radiation source: sealed X-ray tube, 12 x 0.4 mm long-fine focus	2532 reflections with *I* > 2σ(*I*)
plane graphite	*R*_int_ = 0.034
Detector resolution: 6.67 pixels mm^-1^	θ_max_ = 26.5°, θ_min_ = 1.9°
ω–scan rotation method	*h* = −9→9
Absorption correction: integration (*X-RED32*; Stoe & Cie, 2002)	*k* = −11→11
*T*_min_ = 0.956, *T*_max_ = 0.989	*l* = −14→14
8047 measured reflections	

### Refinement

**Table:**

Refinement on *F*^2^	Primary atom site location: structure-invariant direct methods
Least-squares matrix: full	Secondary atom site location: difference Fourier map
*R*[*F*^2^ > 2σ(*F*^2^)] = 0.041	Hydrogen site location: inferred from neighbouring sites
*wR*(*F*^2^) = 0.108	H-atom parameters constrained
*S* = 1.05	*w* = 1/[σ^2^(*F*_o_^2^) + (0.0455*P*)^2^ + 0.127*P*] where *P* = (*F*_o_^2^ + 2*F*_c_^2^)/3
3057 reflections	(Δ/σ)_max_ = 0.001
192 parameters	Δρ_max_ = 0.21 e Å^−^^3^
0 restraints	Δρ_min_ = −0.16 e Å^−^^3^

### Special details

**Table:**

Geometry. All e.s.d.'s (except the e.s.d. in the dihedral angle between two l.s. planes) are estimated using the full covariance matrix. The cell e.s.d.'s are taken into account individually in the estimation of e.s.d.'s in distances, angles and torsion angles; correlations between e.s.d.'s in cell parameters are only used when they are defined by crystal symmetry. An approximate (isotropic) treatment of cell e.s.d.'s is used for estimating e.s.d.'s involving l.s. planes.
Refinement. Refinement of *F*^2^ against ALL reflections. The weighted *R*-factor *wR* and goodness of fit *S* are based on *F*^2^, conventional *R*-factors *R* are based on *F*, with *F* set to zero for negative *F*^2^. The threshold expression of *F*^2^ > σ(*F*^2^) is used only for calculating *R*-factors(gt) *etc*. and is not relevant to the choice of reflections for refinement. *R*-factors based on *F*^2^ are statistically about twice as large as those based on *F*, and *R*- factors based on ALL data will be even larger.

### Fractional atomic coordinates and isotropic or equivalent isotropic displacement parameters (Å^2^)

**Table:**

	*x*	*y*	*z*	*U*_iso_*/*U*_eq_	
C1	0.76186 (18)	0.53676 (14)	0.34535 (12)	0.0411 (3)	
C2	0.75171 (19)	0.51461 (15)	0.46312 (12)	0.0434 (3)	
C3	0.6500 (2)	0.36832 (17)	0.48325 (14)	0.0522 (4)	
H3	0.6401	0.3530	0.5607	0.063*	
C4	0.5636 (2)	0.24556 (17)	0.39194 (16)	0.0584 (4)	
H4	0.4979	0.1488	0.4081	0.070*	
C5	0.5753 (2)	0.26716 (17)	0.27703 (16)	0.0594 (4)	
H5	0.5185	0.1848	0.2151	0.071*	
C6	0.6717 (2)	0.41157 (16)	0.25353 (14)	0.0526 (4)	
H6	0.6766	0.4256	0.1750	0.063*	
C7	0.8491 (2)	0.64107 (17)	0.56952 (13)	0.0522 (3)	
H7A	0.8152	0.6031	0.6396	0.078*	
H7B	0.8031	0.7220	0.5486	0.078*	
H7C	0.9911	0.6787	0.5882	0.078*	
C8	0.87437 (18)	0.69280 (14)	0.31871 (11)	0.0390 (3)	
H8	0.9680	0.7512	0.3977	0.047*	
C9	0.74043 (18)	0.78601 (14)	0.26415 (11)	0.0398 (3)	
C10	0.8251 (2)	0.88159 (16)	0.16893 (12)	0.0454 (3)	
C11	0.7944 (2)	0.76528 (19)	0.06747 (13)	0.0543 (4)	
H11	0.7201	0.7619	−0.0133	0.065*	
C12	0.8816 (2)	0.66815 (18)	0.10214 (13)	0.0528 (4)	
H12	0.8691	0.5884	0.0483	0.063*	
C13	1.00195 (19)	0.69628 (15)	0.23466 (12)	0.0440 (3)	
H13	1.0670	0.6232	0.2537	0.053*	
C14	0.5290 (2)	0.68754 (16)	0.20146 (13)	0.0473 (3)	
C15	0.7433 (2)	0.88992 (15)	0.36219 (12)	0.0438 (3)	
C16	1.0492 (2)	0.97044 (16)	0.23344 (14)	0.0487 (3)	
H16A	1.1033	1.0413	0.1820	0.058*	
H16B	1.0693	1.0283	0.3096	0.058*	
C17	1.1581 (2)	0.86011 (16)	0.25968 (13)	0.0475 (3)	
H17A	1.2439	0.8831	0.3443	0.057*	
H17B	1.2402	0.8707	0.2076	0.057*	
C18	0.7764 (3)	1.0878 (2)	0.0652 (2)	0.0806 (6)	
H18A	0.8949	1.1689	0.1157	0.121*	
H18B	0.6739	1.1264	0.0319	0.121*	
H18C	0.8045	1.0458	−0.0004	0.121*	
N1	0.3655 (2)	0.61425 (17)	0.15462 (14)	0.0698 (4)	
N2	0.7485 (2)	0.96949 (16)	0.43909 (13)	0.0622 (4)	
O1	0.71183 (17)	0.97361 (13)	0.13594 (10)	0.0617 (3)	

### Atomic displacement parameters (Å^2^)

**Table:**

	*U*^11^	*U*^22^	*U*^33^	*U*^12^	*U*^13^	*U*^23^
C1	0.0353 (6)	0.0387 (7)	0.0463 (7)	0.0129 (5)	0.0108 (5)	0.0023 (5)
C2	0.0361 (6)	0.0444 (7)	0.0498 (7)	0.0165 (5)	0.0125 (5)	0.0072 (6)
C3	0.0439 (7)	0.0532 (8)	0.0595 (9)	0.0167 (6)	0.0181 (6)	0.0173 (7)
C4	0.0450 (8)	0.0424 (8)	0.0802 (11)	0.0101 (6)	0.0173 (7)	0.0138 (7)
C5	0.0509 (8)	0.0404 (7)	0.0717 (10)	0.0082 (6)	0.0106 (7)	−0.0054 (7)
C6	0.0512 (8)	0.0448 (7)	0.0531 (8)	0.0117 (6)	0.0133 (6)	−0.0010 (6)
C7	0.0558 (8)	0.0546 (8)	0.0455 (8)	0.0200 (7)	0.0165 (6)	0.0070 (6)
C8	0.0364 (6)	0.0384 (6)	0.0374 (6)	0.0114 (5)	0.0091 (5)	0.0003 (5)
C9	0.0366 (6)	0.0418 (7)	0.0387 (6)	0.0130 (5)	0.0115 (5)	0.0020 (5)
C10	0.0438 (7)	0.0520 (8)	0.0424 (7)	0.0192 (6)	0.0151 (6)	0.0116 (6)
C11	0.0471 (8)	0.0721 (10)	0.0357 (7)	0.0136 (7)	0.0132 (6)	0.0031 (7)
C12	0.0504 (8)	0.0570 (8)	0.0455 (8)	0.0110 (7)	0.0203 (6)	−0.0073 (6)
C13	0.0405 (7)	0.0437 (7)	0.0475 (7)	0.0141 (6)	0.0164 (6)	0.0002 (6)
C14	0.0415 (7)	0.0510 (8)	0.0486 (7)	0.0171 (6)	0.0143 (6)	0.0012 (6)
C15	0.0472 (7)	0.0431 (7)	0.0436 (7)	0.0189 (6)	0.0160 (6)	0.0070 (6)
C16	0.0452 (7)	0.0456 (7)	0.0518 (8)	0.0110 (6)	0.0184 (6)	0.0071 (6)
C17	0.0378 (7)	0.0513 (8)	0.0491 (7)	0.0111 (6)	0.0154 (6)	0.0038 (6)
C18	0.0877 (14)	0.0894 (13)	0.0880 (13)	0.0478 (11)	0.0411 (11)	0.0502 (11)
N1	0.0413 (7)	0.0762 (9)	0.0777 (10)	0.0141 (7)	0.0108 (6)	−0.0124 (8)
N2	0.0799 (9)	0.0595 (8)	0.0570 (8)	0.0357 (7)	0.0249 (7)	0.0036 (6)
O1	0.0616 (7)	0.0731 (7)	0.0663 (7)	0.0370 (6)	0.0273 (5)	0.0331 (6)

### Geometric parameters (Å, °)

**Table:**

C1—C6	1.3955 (19)	C10—O1	1.4160 (17)
C1—C2	1.4019 (19)	C10—C11	1.501 (2)
C1—C8	1.5153 (17)	C10—C16	1.5372 (19)
C2—C3	1.3923 (19)	C11—C12	1.321 (2)
C2—C7	1.5068 (19)	C11—H11	0.93
C3—C4	1.378 (2)	C12—C13	1.494 (2)
C3—H3	0.93	C12—H12	0.93
C4—C5	1.372 (2)	C13—C17	1.5417 (19)
C4—H4	0.93	C13—H13	0.98
C5—C6	1.382 (2)	C14—N1	1.1355 (19)
C5—H5	0.93	C15—N2	1.1371 (18)
C6—H6	0.93	C16—C17	1.535 (2)
C7—H7A	0.96	C16—H16A	0.97
C7—H7B	0.96	C16—H16B	0.97
C7—H7C	0.96	C17—H17A	0.97
C8—C13	1.5551 (18)	C17—H17B	0.97
C8—C9	1.5816 (17)	C18—O1	1.408 (2)
C8—H8	0.98	C18—H18A	0.96
C9—C15	1.4776 (18)	C18—H18B	0.96
C9—C14	1.4804 (18)	C18—H18C	0.96
C9—C10	1.5794 (18)		
			
C6—C1—C2	118.87 (12)	C11—C10—C16	109.89 (12)
C6—C1—C8	120.32 (12)	O1—C10—C9	104.47 (10)
C2—C1—C8	120.77 (11)	C11—C10—C9	104.90 (11)
C3—C2—C1	118.44 (13)	C16—C10—C9	107.10 (11)
C3—C2—C7	118.43 (13)	C12—C11—C10	114.56 (12)
C1—C2—C7	123.11 (12)	C12—C11—H11	122.7
C4—C3—C2	121.98 (14)	C10—C11—H11	122.7
C4—C3—H3	119.0	C11—C12—C13	114.81 (13)
C2—C3—H3	119.0	C11—C12—H12	122.6
C5—C4—C3	119.50 (14)	C13—C12—H12	122.6
C5—C4—H4	120.3	C12—C13—C17	106.54 (12)
C3—C4—H4	120.3	C12—C13—C8	111.97 (11)
C4—C5—C6	119.87 (14)	C17—C13—C8	105.91 (10)
C4—C5—H5	120.1	C12—C13—H13	110.7
C6—C5—H5	120.1	C17—C13—H13	110.7
C5—C6—C1	121.31 (15)	C8—C13—H13	110.7
C5—C6—H6	119.3	N1—C14—C9	178.43 (15)
C1—C6—H6	119.3	N2—C15—C9	178.70 (15)
C2—C7—H7A	109.5	C17—C16—C10	110.08 (11)
C2—C7—H7B	109.5	C17—C16—H16A	109.6
H7A—C7—H7B	109.5	C10—C16—H16A	109.6
C2—C7—H7C	109.5	C17—C16—H16B	109.6
H7A—C7—H7C	109.5	C10—C16—H16B	109.6
H7B—C7—H7C	109.5	H16A—C16—H16B	108.2
C1—C8—C13	115.60 (10)	C16—C17—C13	108.74 (11)
C1—C8—C9	114.62 (10)	C16—C17—H17A	109.9
C13—C8—C9	107.25 (10)	C13—C17—H17A	109.9
C1—C8—H8	106.2	C16—C17—H17B	109.9
C13—C8—H8	106.2	C13—C17—H17B	109.9
C9—C8—H8	106.2	H17A—C17—H17B	108.3
C15—C9—C14	106.69 (11)	O1—C18—H18A	109.5
C15—C9—C10	109.45 (11)	O1—C18—H18B	109.5
C14—C9—C10	108.55 (11)	H18A—C18—H18B	109.5
C15—C9—C8	110.69 (10)	O1—C18—H18C	109.5
C14—C9—C8	112.91 (11)	H18A—C18—H18C	109.5
C10—C9—C8	108.49 (10)	H18B—C18—H18C	109.5
O1—C10—C11	114.96 (12)	C18—O1—C10	116.48 (12)
O1—C10—C16	114.58 (12)		
			
C6—C1—C2—C3	−0.63 (19)	C14—C9—C10—C11	−57.83 (14)
C8—C1—C2—C3	−178.64 (12)	C8—C9—C10—C11	65.22 (13)
C6—C1—C2—C7	178.11 (13)	C15—C9—C10—C16	69.33 (14)
C8—C1—C2—C7	0.10 (19)	C14—C9—C10—C16	−174.58 (11)
C1—C2—C3—C4	1.4 (2)	C8—C9—C10—C16	−51.54 (13)
C7—C2—C3—C4	−177.44 (13)	O1—C10—C11—C12	−172.24 (12)
C2—C3—C4—C5	−0.8 (2)	C16—C10—C11—C12	56.73 (16)
C3—C4—C5—C6	−0.6 (2)	C9—C10—C11—C12	−58.09 (15)
C4—C5—C6—C1	1.3 (2)	C10—C11—C12—C13	−2.60 (18)
C2—C1—C6—C5	−0.7 (2)	C11—C12—C13—C17	−57.14 (16)
C8—C1—C6—C5	177.32 (13)	C11—C12—C13—C8	58.22 (17)
C6—C1—C8—C13	−39.45 (17)	C1—C8—C13—C12	85.22 (14)
C2—C1—C8—C13	138.53 (12)	C9—C8—C13—C12	−44.02 (14)
C6—C1—C8—C9	86.10 (15)	C1—C8—C13—C17	−159.04 (11)
C2—C1—C8—C9	−95.92 (14)	C9—C8—C13—C17	71.72 (12)
C1—C8—C9—C15	95.40 (13)	O1—C10—C16—C17	−177.48 (11)
C13—C8—C9—C15	−134.81 (11)	C11—C10—C16—C17	−46.25 (15)
C1—C8—C9—C14	−24.13 (15)	C9—C10—C16—C17	67.16 (14)
C13—C8—C9—C14	105.66 (12)	C10—C16—C17—C13	−10.53 (15)
C1—C8—C9—C10	−144.50 (11)	C12—C13—C17—C16	61.71 (14)
C13—C8—C9—C10	−14.70 (13)	C8—C13—C17—C16	−57.67 (14)
C15—C9—C10—O1	−52.60 (13)	C11—C10—O1—C18	−74.84 (18)
C14—C9—C10—O1	63.49 (13)	C16—C10—O1—C18	53.89 (18)
C8—C9—C10—O1	−173.47 (10)	C9—C10—O1—C18	170.76 (14)
C15—C9—C10—C11	−173.91 (11)		

### Hydrogen-bond geometry (Å, °)

**Table:**

*D*—H···*A*	*D*—H	H···*A*	*D*···*A*	*D*—H···*A*
C12—H12···N1^i^	0.93	2.70	3.509 (3)	146
C7—H7C···Cg1^ii^	0.96	2.84	3.688 (2)	146

Symmetry codes: (i) −*x*+1, −*y*+1, −*z*; (ii) −*x*+2, −*y*+1, −*z*+1.
